# Novel mechanism of the COVID-19 associated coagulopathy (CAC) and vascular thromboembolism

**DOI:** 10.1038/s44298-023-00003-3

**Published:** 2023-10-18

**Authors:** Mahavir Singh, Sathnur Pushpakumar, Yuting Zheng, Irina Smolenkova, Oluwaseun E. Akinterinwa, Bana Luulay, Suresh C. Tyagi

**Affiliations:** 1 Department of Physiology, University of Louisville School of Medicine, Louisville, KY 40202, USA.; 2 These authors contributed equally: Mahavir Singh, Sathnur Pushpakumar.

## Abstract

Previous studies from our laboratory revealed that SARS-CoV-2 spike protein (SP) administration to a genetically engineered model expressing the human angiotensin-converting enzyme 2; ACE2 receptor (i.e., hACE2 humanized mouse) mimicked the coronavirus disease-19 (COVID-19) pathology. In humans the cause of high morbidity, and mortality is due to ‘*cytokine-storm*’ led thromboembolism; however, the exact mechanisms of COVID-19 associated coagulopathy (CAC) have yet to be discovered. Current knowledge suggests that CAC is distinct from the standard coagulopathy, in that the intrinsic and extrinsic thrombin-dependent coagulation factors, and the pathway(s) that are common to coagulopathy, are not recruited by SARS-CoV-2. Findings from patients revealed that there is little change in their partial thromboplastin, or the prothrombin time coupled with a significant decline in platelets. Further, there appears to be an endothelial dysfunction during COVID-19 suggesting an interaction of the endothelia with immune cells including neutrophils. There are also reports that inflammatory NGAL is elevated during COVID-19. Furthermore, the levels of NPT are also increased indicating an increase in inflammatory M1 macrophage iNOS which sequesters BH4; an essential enzyme co-factor that acts as a potent antioxidant thus causing damage to endothelia. SARS-CoV-2 entry into the host cells is facilitated by a co-operative action between TMPRSS2 and the main ACE2 receptor. Interestingly, after infection ADAMTS13; a von Willebrand factor; VWF cleaving enzyme is found to be decreased. Based on these facts, we hypothesize that vascular thromboembolism is associated with serine and metalloproteinase, and in that context, we opine that inhibition of iNOS might help mitigate COVID-19 harmful effects. To test this hypothesis, we administered SP to the hACE2 mice that were subsequently treated with amino guanidine (AG; a potent inhibitor of glycoxidation, lipoxidation and oxidative vicious cycles). Our results revealed increase in TMPRSS2, and NGAL by SP but treatment with AG mitigated their levels. Similarly, levels of MMP-2, and −9 were increased; however, AG treatment normalized these levels. Our findings suggest that occurrence of CAC is influenced by TMPRSS2, ADAMTS13, NGAL and MMP- 2, and −9 factors, and an intervention with iNOS blocker helped mitigate the CAC condition in experimental settings.

## INTRODUCTION

The severe acute respiratory syndrome coronavirus 2; SARS-CoV-2 is responsible for coronavirus disease^1,2^. COVID-19 induced coagulopathy appears to be different from the traditional coagulopathy in many ways since it meets the criteria for the relatively newly defined entity of sepsis-induced coagulopathy (SIC) that is characterized, and quantified according to reduced platelet counts, increased international normalized ratio (INR), and higher organ dysfunction in SARS-CoV-2 patients^3^. The findings during the last few years after COVID-19 pandemic have suggested that SARS-CoV-2 has a connection with various blood disorders, including a higher risk of clot formation and sometimes bleeding problems in acutely infected patients^4–7^. During infection, the frequently observed micro- and macro- thrombotic events are due to the perpetuation of a state of hypercoagulability that has been termed as the COVID-19-associated coagulopathy (CAC) and, in fact, it is different from the regular clotting problems^8^. The so-called CAC represents a key aspect for the development of multi-organ damage in patients. In CAC the changes are represented by high levels of D-dimer and fibrinogen; however, CAC also has some common features with disseminated intravascular coagulation (DIC) and SIC, but there are differences between these clinical observations. It appears that the pathogenesis of CAC is more complex and is influenced by an interconnection between the inflammatory system and coagulation, in the phenomenon of immuno-thrombosis and thromboinflammation. In CAC many factors come into play including the neutrophils, inflammatory cytokines, complement system as well as fibrinolytic system. Finally, changes in platelet function coupled with endothelial dysfunction also play roles in CAC. Though we are still piecing together exactly how it happens, several factors such as problems with endothelial lining, inflammation, and an intense immune system response affects in raising the risk of clotting. Moreover, in severely acute infection, the immune system can go off the track. This can lead to additional issues with a part of the immune system called the “complement system,” which can also damage blood vessels making clotting problems worse^9^.

When platelets get activated, they stick together and release chemicals that promote inflammation. This makes blood clot formation more easily. In regular clotting, the making of “thrombin” helps turn “fibrinogen” into “fibrin” the material that makes up clots. Conditions like hemophilia, where some clotting factors are missing, or DIC, where too much of this process happens, lead to standard clotting problems. So, the main differences between CAC and regular clotting problems lie in how they happen. In regular clotting problems, making of thrombin is important for clotting to occur but in CAC, the risk of blood clots arises from a mix of inflammation, problems with blood vessel lining, platelet activation, and issues with the immune system’s response^9^. The “spike protein” or SP also interacts with the clotting process. This contributes to the unique clotting situation seen in CAC. Understanding these aspects of CAC is crucial for devising care for COVID-19 patients, especially for people with weakened immune systems like diabetics, who face clotting-related problems more often than those without diabetes^10–16^.

Briefly, the coronaviruses that cause severe acute respiratory syndrome (SARS), such as SARS-CoV, MERS-CoV (responsible for the middle east respiratory syndrome), and SARS-CoV-2, need transmembrane serine protease 2 (TMPRSS2) for their entry into the host cells. Subsequent studies have also shown that SARS-CoV-2 requires the angiotensin-converting enzyme 2 (ACE2) as the main receptor together with TMPRSS2 for infecting the host cells productively^17–19^. SARS-CoV-2 is a single-stranded ribonucleic acid (RNA) virus that encodes a variety of proteins, including 4 structural proteins such as (1) Membrane/Matrix (M), (2) Envelope (E), and (3) Spike (S) proteins that assemble around (4) Nucleocapsid (N) and its RNA. As mentioned above, the virus infects host cells upon binding to ACE2 receptor, and subsequent action of proteases, including the transmembrane protease serine 2 (TMPRSS2). The virus exhibits a high infectious rate and can provoke a wide array of symptoms beginning with the “cytokine storm”^20,21^. The recent pandemic is the third outbreak due to a highly pathogenic β-coronavirus in only just two decades. Thus, there is a tremendous need to acquire in-depth knowledge of the virus infection cycle, as well as the cellular and molecular pathways that are involved in the viral replication and mounting of the innate and adaptive immune responses in the host. We believe that answers to these fundamental questions will help us in the development of safer, and efficient therapeutics and pan-β-coronavirus vaccines against emerging SARS-CoV-2 variants and their subvariants of concern^22^. ACE turns angiotensin I into angiotensin II, which has multiple effects throughout our body such as increase in blood pressure. Angiotensin I is cleaved by angiotensin-converting enzyme (ACE) to produce angiotensin II, and then Angiotensin II binds to its receptors and exerts its effects in the brain, kidney, adrenal, vascular wall, and the heart. Although SARS-CoV-2 spike protein (SP) masks the ACE2, but it increases the availability of angiotensin (Ang II; 1–8) and decreases the Ang (1–7), suggesting a role in hypertension^23–26^. Although COVID-19 has been linked to orthostatic tachycardia (OT), again the mechanisms are largely unknown. The heart rate of the patient increases by ~30 beats/min; however, the blood flow to the brain decreases that causes hypoperfusion, and vascular contributions to cognitive impairment and dementia (VCID). This also suggests a role of vascular coagulopathy, and thrombosis for the decrease in the blood flow to brain^27^. Since so many complications are associated with COVID-19 including the vascular coagulopathy/thromboembolism, endothelial dysfunction, stiffness, fibrosis, and extracellular matrix (ECM) fragmentation^9,28–36^, but the underlying mechanisms of these events are unclear.

The ongoing research in our laboratory is based on our previous studies that have shown activation of inflammatory M1 macrophages with renal infiltrates in the hACE2 mice administered with SP, intranasally^37^. In COVID-19 patients there is an elevated level of neopterin (NPT)^38,39^. Because NPT is generated by activated pro-inflammatory macrophages (M1) in response to COVID-19 via inducible nitric oxide synthase (iNOS), tetrahydrobiopterin (BH4), and peroxinitrite (ONOO-) along with activation of proteinases. We are of the opinion that iNOS generates NPT and decreases BH4, eNOS activity and a disintegrin and metalloproteinase thrombospondin 13 domain (ADAMTS13), anti-thrombosis/anti-coagulant but at the same time activates urinary neutrophil gelatinase associated lipocalin2 (NGAL2, pro-thrombosis/pro-coagulant), and transmembrane serine proteinase S2 (TMPTSS2, proteolytic factor processing of COVID-19) and matrix metalloproteinases (MMPs), leading to renal dysfunction and failure^18,40–43^. NGAL and FGF23 (vascular hypertrophic factor) are high and ADAMTS13 is low post COVID-19 sequelae^44–48^. Again, the recognition of endothelial dysfunction within the realm of COVID-19 is emerging as a pivotal and defining aspect of SARS-CoV-2 infection, and its post infection implications especially against the background of the intricate interplay between endothelial cells and immune constituents, exemplified by the dynamic interaction with neutrophils in the context of thromboembolism, assumes paramount significance. This alliance between endothelial cells lining and immune effector molecules that our work has reported earlier prompts a series of inquiries that underscore the pivotal role of endothelial cells in the intricate landscape of thromboembolic events^37^. A further deeper exploration into the mechanisms underpinning endothelial dysfunction can unravel its contribution to the disruption of coagulation equilibrium, particularly within the intricate milieu of CAC. This knowledge will enhance our comprehension of the pathophysiological mechanisms at play but also to pave the way for the identification of strategic interventions aimed at safeguarding our patients during the throes of acute infection. Hence, by delving into the nuanced of how endothelial dysfunction precipitates coagulation dysregulation, particularly within the context of COVID-19 related coagulopathic manifestations, a potential roadmap might emerge via an understanding of the molecular interactions that could hold the promise of unveiling therapeutic avenues designed to shield afflicted patients during the critical phase of acute infection^49^.

Interestingly, CAC in glomerular capillaries-microvascular wall, the interaction between macrophages, neutrophil and immune cells, causes build-up layers of damage endothelial via iNOS, NGAL2, eNOS, and ADAMTS13, sets the stage of pro-thrombotic and pro-coagulant processes. This also contributes to focal glomerulosclerosis lesions in COVID-19 patients^50,51^. The levels of transmembrane (TMPRSS2), EMMPRIN (CD147) and ECM proteinases are elevated post COVID-19^18^. These proteinases are associated with collagen/elastin breakdown during renal glomerular remodeling. However, because the turnover of collagen is rapid than elastin, the degraded elastin is replaced by collagen, causing fibrosis, stiffness, and thickening of the basement membrane in the glomeruli, instigating impaired glomerular filtration rate (GFR)^52^. Therefore, an increase in M1 macrophages iNOS decreases BH4 bioavailability to eNOS, causing glomerular capillary microvascular endothelial dysfunction. The oxidative peroxinitrite (ONOO-) activates NGAL2 and FGF23 and decrease in ADAMTS13^53,54^. The transmembrane serine (TMPRSS2), EMMPRIN and MMPs/TIMPs are activated, leading to renal dysfunction. Interestingly, there appears to be evidence of fibrosis with hypertrophy in the glomeruli of hACE2 mice administered with SP within 4 weeks post COVID-19 sequelae, as expected, since this will suggest that there is acute kidney injury (AKI) with preserved glomerular filtration rate (GFR), that can be reversed by iNOS blocker^37^. However, if there is chronic thickening of the basement membrane, post COVID-19, this will suggest that there is chronic kidney disease (CKD). In that situation an inhibitor of proteinases, such as TMPRSS2 will be able to mitigate the COVID-19 induced CKD. This is important and novel in the sense that post COVID-19 morbidities, and mortalities can be halted with a TMPRSS2 inhibitor.

Further, in the landscape of biomarkers linked to endothelial dysfunction, the circulating inflammatory coagulation markers assume a pivotal role, notably the fibrin(ogen), D-dimer, P-selectin, and von Willebrand Factor (VWF). These biomarkers, in their close relationship with endothelial cells, platelets, and erythrocytes, contribute significantly to the pathological progression observed in acute COVID-19 cases^55^. For example, the fibrin(ogen) and D-dimer are indicative of ongoing coagulation and fibrinolysis processes, exhibiting a dynamic connection with MMP-2, MMP-9, and MMP-13^56^. These MMPs can potentially modulate the stability of blood clots, thereby impacting both clot formation and dissolution. The interplay between these biomarkers might amplify the coagulopathic tendencies observed in severe COVID-19 patients, thus accentuating the pro-thrombotic environment. Interestingly, the Von Willebrand Factor (VWF) and P-selectin are integral to platelet adhesion and aggregation, and intersect with ADAMTS-13, a critical regulator of VWF cleavage^57,58^. Thus, a dysregulation of ADAMTS-13 in COVID-19 could lead to increased VWF activity, thereby fostering the microvascular thrombosis. This interaction, coupled with the intricate role of MMPs, might contribute to the endothelial activation and damage central to the disease pathogenesis. On the other hand, the renal NGAL, is a biomarker of kidney injury, and that usually reflects the broader systemic impact of inflammation and coagulation in COVID-19 patients^59^. Its interplay with MMP-7 or matrilysins (it was originally described as PUMP-1; putative uterine metalloprotease-1), and was long considered a third member of the stromelysin family, although it appeared only distantly related to the other stromelysins, and later dubbed MMP-7, a possible reference to ion transporters, underscores the systemic implications of electrolyte imbalance in severely ill patients, potentially affecting both coagulation and vascular health^60,61^. Furthermore, TMPRSS2, an enzyme facilitating viral entry, adds another layer to this complex narrative. Its interplay with these biomarkers might signify a feedback loop where the virus-induced dysregulation contributes to coagulopathic tendencies, potentially mediated by the interplay of endothelial cells, platelets, and erythrocytes^62–66^. In a nutshell, the interaction between fibrin(ogen), D-dimer, P-selectin, VWF, and biomarkers like MMPs, ADAMTS-13, renal NGAL, PUMP, and TMPRSS2 intertwine within the context of acute COVID-19. This intricate interplay encompasses endothelial dysfunction, platelet aggregation, coagulopathy, and systemic impact, collectively contributing to the complex pathogenesis observed in severe cases. Understanding these connections will aid not only in deciphering disease mechanisms but also in the identification of potential avenues for therapeutic intervention for the patients.

In the intricate landscape of biomarkers linked to endothelial dysfunction, certain circulating inflammatory coagulation biomarkers assume a pivotal role, notably the fibrin(ogen), D-dimer, P-selectin, and von Willebrand Factor (VWF). These biomarkers, in their close relationship with endothelial cells, platelets, and erythrocytes, contribute significantly to the pathological progression observed in acute COVID-19 cases. For example, the fibrin(ogen) and D-dimer are indicative of ongoing coagulation and fibrinolysis processes, exhibiting a dynamic connection with MMP-2, MMP-9, and MMP-13. These MMPs can potentially modulate the stability of blood clots, thereby impacting both clot formation and dissolution. The interplay between these biomarkers might amplify the coagulopathic tendencies observed in severe COVID-19 patients, thus accentuating the pro-thrombotic environment. Interestingly, the Von Willebrand Factor (VWF) and P-selectin are integral to platelet adhesion and aggregation, and intersect with ADAMTS-13, a critical regulator of VWF cleavage. Thus, a dysregulation of ADAMTS-13 in COVID-19 could lead to increased VWF activity, thereby fostering the microvascular thrombosis. This interaction, coupled with the intricate role of MMPs, might contribute to the endothelial activation and damage central to the disease pathogenesis. On the other hand, the renal NGAL, is a biomarker of kidney injury, and that usually reflects the broader systemic impact of inflammation and coagulation in COVID-19 patients. Its interplay with MMP-7 or matrilysins (it was originally described as putative uterine metalloprotease-1 (PUMP-1) in 1988, and was long considered a third member of the stromelysin family, although it appeared only distantly related to the other stromelysins, and later dubbed MMP-7, a possible reference to ion transporters, underscores the systemic implications of electrolyte imbalance in severely ill patients, potentially affecting both coagulation and vascular health. Furthermore, the TMPRSS2, an enzyme facilitating viral entry, adds another layer to this complex narrative. Its interplay with these biomarkers might signify a feedback loop where the virus-induced dysregulation contributes to coagulopathic tendencies, potentially mediated by the interplay of endothelial cells, platelets, and erythrocytes. In summary, the interaction between fibrin(ogen), D-dimer, P-selectin, VWF, and the mentioned biomarkers like MMPs, ADAMTS-13, renal NGAL, PUMP, and TMPRSS2 intertwines within the context of acute COVID-19. This intricate interplay encompasses endothelial dysfunction, platelet aggregation, coagulopathy, and systemic impact, collectively contributing to the complex pathogenesis observed in severe cases. Understanding these connections will aid not only in deciphering disease mechanisms but also in identifying potential avenues for therapeutic interventions.

## RESULTS

### Effect of the SARS-CoV-2 spike protein (SP) administration in the kidney

**Fig. 1:**
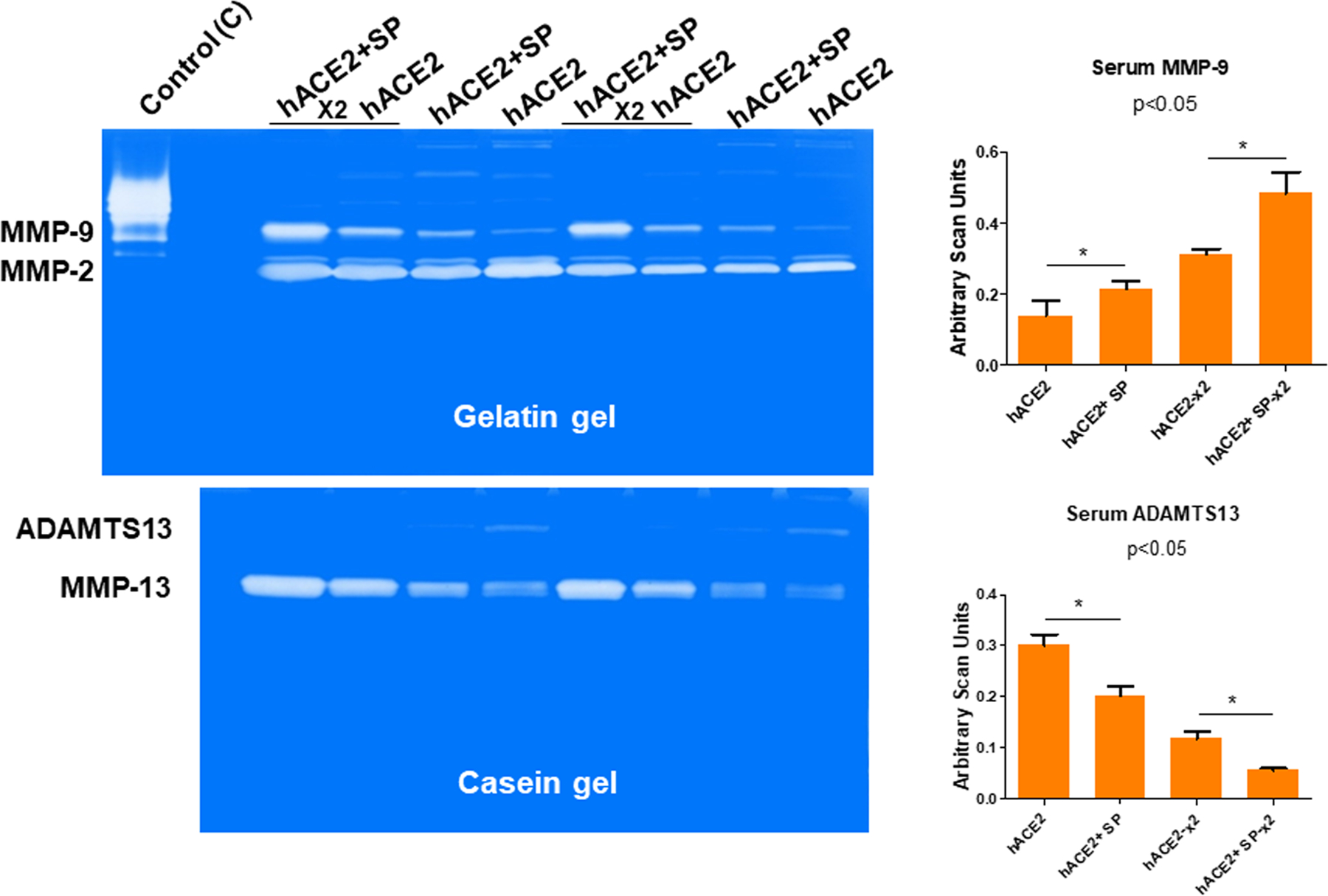
Determination of the matrix metalloproteinase (MMPs) activities. The zymographic analysis of the sera samples collected from mice that were either administered with or without SARS-CoV-2 spike protein (SP) intra-renally into the cortex. The samples were loaded on to the gelatin (upper panel) and casein (lower panel) gels. Lanes are labeled as: hACE2 (no SP), and hACE2+SP (with SP), samples were loaded in duplicates with the 2× concentration and 1× concentrations respectively. Collagenase was used as a standard control. Relative levels of the gelatinase (MMP-2 and MMP-9) and the caseinase; MMP-13 and ADAMTS13) activities are shown. The bar graphs are representations of the scan units from the gels, represented as the Mean ± SD, *n* = 4, SP, * *p* < 0.05.

To determine whether the SARS-CoV-2 spike protein (SP) injures the kidney, we injected the SP into the renal cortex of hACE2 mice and monitored the levels of injury by measuring the activities of key indicator molecules such as the levels of serum MMP-9 after the SP administration. The results suggested that SP causes the metalloproteinases (MMPs) activation in the hACE2 mice. For example, the activity of MMP-9 was found to be doubled when the x2 concentration was used. However, the levels of ADAMTS13 were decreased. It seems that it could serve as a marker of for the venous thromboembolism (VTE) as seen after the SP injection in the hACE2 mice, in comparison to the untreated mice (Fig. 1). The ADAMTS13 is a disintegrin and metalloproteinase with a thrombospondin type 1 motif, member 13 antigen and in the human SARS-CoV-2 patients it has been recently shown to be decreased in comparison with the controls (non-infected subjects)^42^.

### The spike protein (SP) of SARS-CoV-2 leads to increased levels of renal neutrophil gelatinase associated lipocalin (NGAL)

**Fig. 2:**
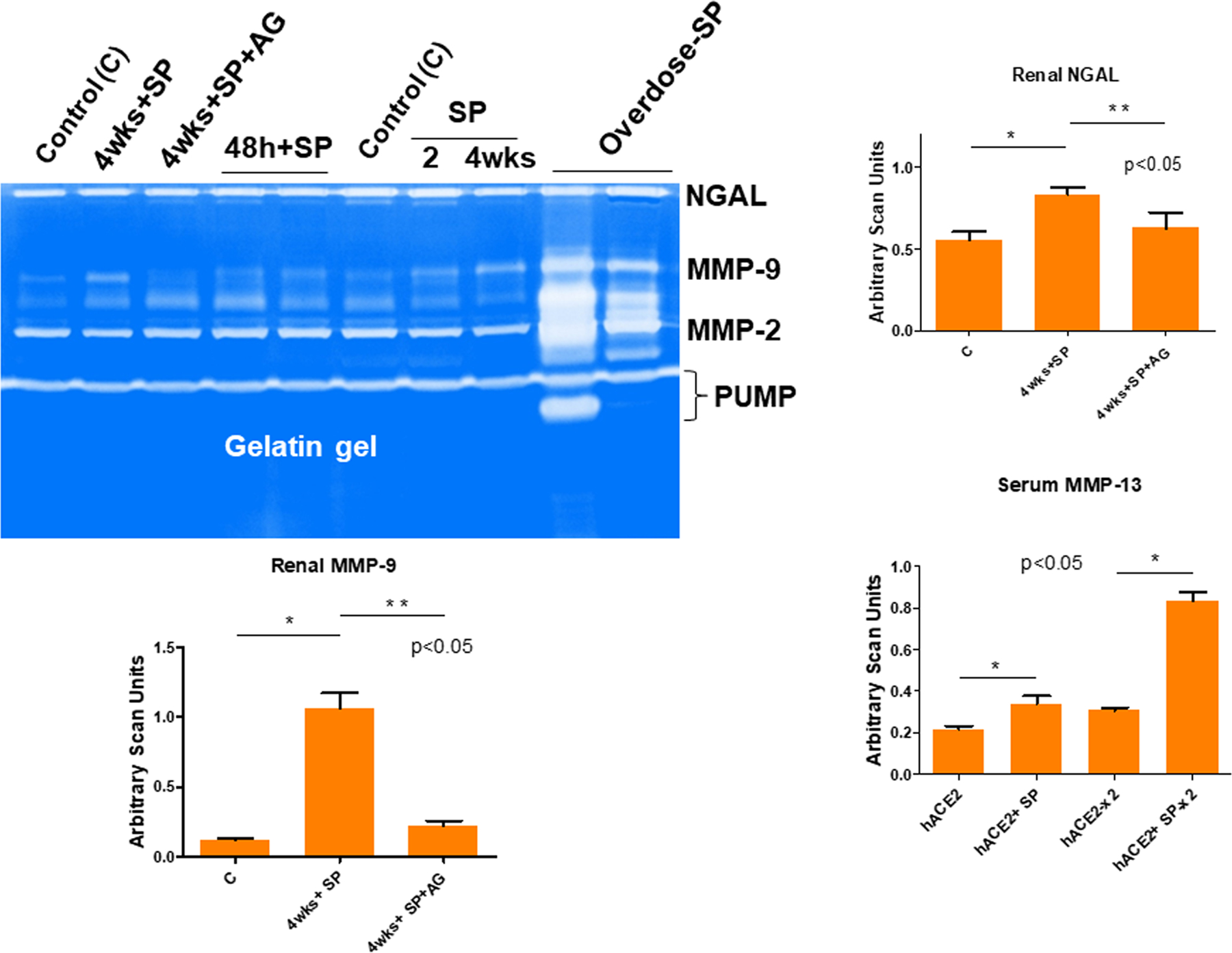
Estimation of the NGAL and MMP-9 levels. The renal cortex MMP-9 and NGAL activities were determined by gelatin (left upper gel panel) using the gel zymography. Lanes are labeled as: Control (C) i.e., the hACE2 mice (no SP), 4wks+SP. An intra-renal (into the cortex) injection was used for administering the SARS-CoV-2 spike protein (SP), and the samples were collected at 2, 4 wks, and at 4wks+SP + AG (treatment and untreated with aminoguanidine; AG an iNOS inhibitor). The sera samples from mice (hACE2 1×, 2× and hACE2+SP 1×, 2×) were used for MMP-13 quantitation using the zymography (bar graph). The bar graphs are representations of the scan units from the gel’s panels, Mean ± SD, *n* = 4, * and ** *p* < 0.05. PUMP putative/truncated metalloproteinase.

In fact, the MMP-13 and ADAMTS13 are very strong caseinases as compared to the relative gelatinases’ activities. Further, the MMP-13 is associated with growth arrest DNA damage (GADD45/MMP13) and may be responsible for causing the COVID-19 associated coagulopathy (CAC) in susceptible human subjects. Our results clearly show that the MMP-13 activity is increased by SP administration in the hACE2 mice when compared with untreated hACE2 mice (Fig. 2). Further, to determine specific kidney injury by the SP, we measured renal cortex levels of gelatinases with the help of zymography technique. The findings revealed that there was a robust increase in the renal NGAL, and MMP-9 by SP 4 wks post injection/administration. Such findings have also been reported by others in the patients suffering from with COVID-19 because it is thought to be associated with histopathologic injury, loss of kidney function, and severity of patient outcomes^59,67,68^. Interestingly, the treatment with aminoguanidine (AG); an iNOS inhibitor mitigated this increase in NGAL levels, as well as MMP-9.

### The renal transmembrane serine protease 2 (TMPRSS2) levels post administration of spike protein (SP)

**Fig. 3:**
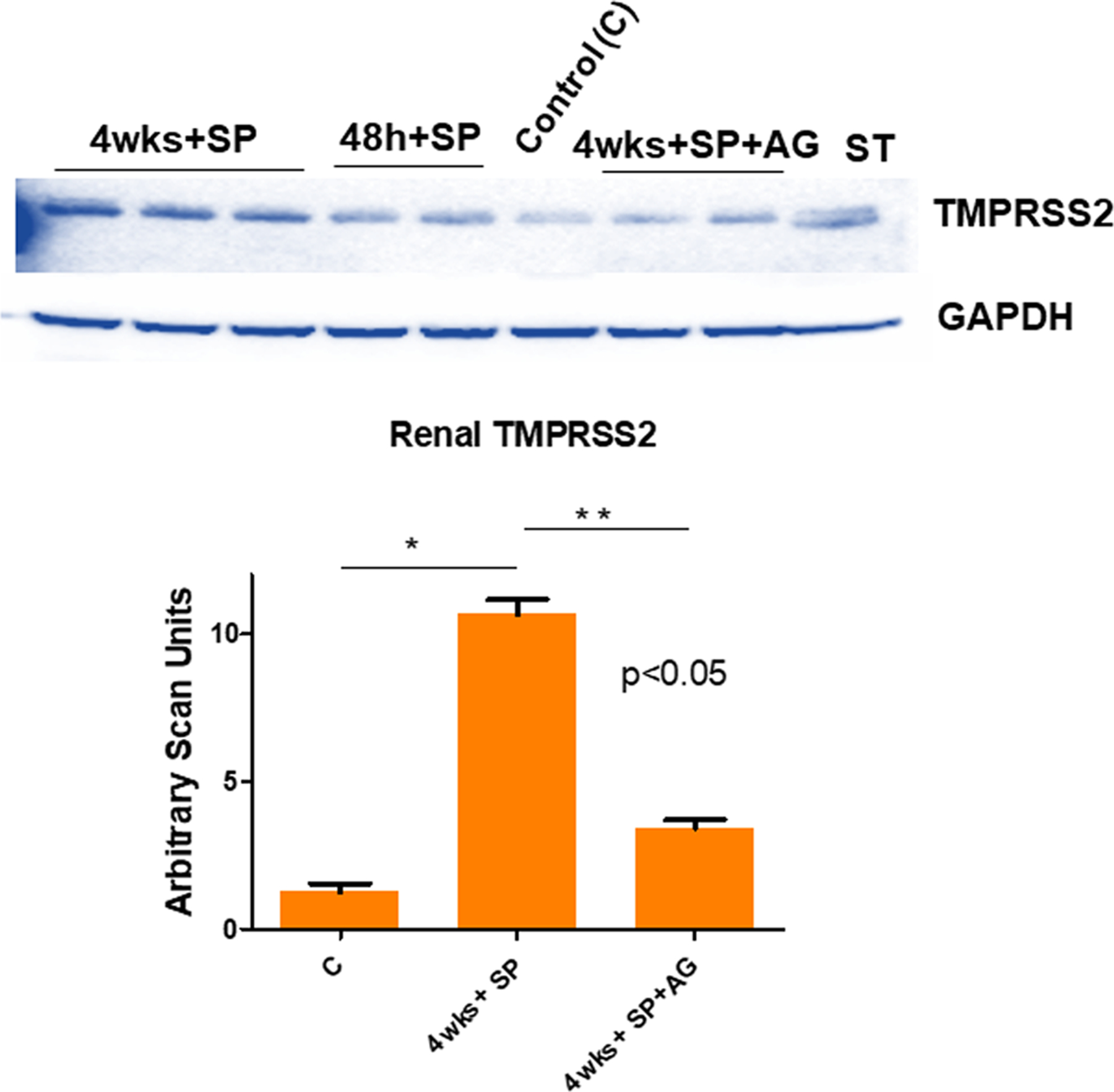
Levels of TMPRSS2 in the renal cortex. A representative immunoblotting showing the TMPRSS2 levels. Lanes are labeled as: Control (C) i.e., the hACE2 mice, 4 wks + SP, treatment with intrarenal injection into the cortex of the SARS-CoV-2 spike protein (SP) for 48 h., and 4 wks, 4wks+SP + AG, treatment with aminoguanidine (AG; iNOS inhibitor). The bar graph is representing the scan units from the gel’s panel, Mean ± SD, *n* = 4, * and ** *p* < 0.05.

To determine whether the proprotein convertase/secretase transmembrane serine proteinase domain S2 is increased post SP administration, we measured the levels of TMPRSS2 in hACE2 mice treated with SP by Western blot analysis. Our results suggested a robust increase in TMPRSS2 by SP administration. It is worth mentioning here that among many genetic markers, the G carriers of *TMPRSS2* (rs2070788) have an increased risk of severe COVID-19 outcomes as compared to those with A/A genotype^69^. Interestingly, the inhibition of inflammatory M1 iNOS by AG mitigated this increase in TMPRSS2 by SP (Fig. 3). We surmise that the potential role(s) of the iNOS inhibitor and that of the TMPRSS2 inhibitor in the renal microvascular remodeling, could prove to be novel as also demonstrated by other investigators that during glomerulosclerosis in COVID-19 and its mitigation by TMPRSS2 inhibitor(s) is indeed therapeutically novel concept^18^. The role of iNOS in endothelial dysfunction especially after COVID-19, is also of high importance. Although, tests of renal function can be used to assess overall renal function by direct measurement or estimation of the glomerular filtration rate (GFR); however, it has been proposed by our group in the past that the treatment with iNOS inhibitor and with doxycycline to inhibit the proteinases and reversal of the renovascular endothelial dysfunction are therapeutically a simple and novel approach^70^. In that context, the use of iNOS knockout (iNOSKO) models, to study the COVID-19 sequelae, can prove a rewarding approach too since mitigation of systemic remodeling that is invariably induced by COVID-19, thus making use of the iNOSKO mice might be a good idea. The measurements of urinary NGAL2, indicative of primary focal segmental glomerulosclerosis lesions by in-gel-gelatinase zymographic assays, is again a novel concept^43,50,51^. Furthermore, renal cortex TMPRSS2 activity (in the membrane fraction) could also be measured by serine proteinase assay, such as the “in gel” plasminogen zymography.

## DISCUSSION

Coronaviruses constitute a large virus family that has hundreds of members. They are generally responsible for causing a mild upper-respiratory tract illness, like the common cold. However, since the turn of the century, new coronaviruses have jumped the species barriers, and hence led to serious morbidity, and increased mortality globally. For example, the SARS coronavirus (SARS-CoV) caused severe acute respiratory syndrome (SARS) in 2002 but it disappeared by 2004^71–73^. The MERS coronavirus (MERS-CoV) led to the middle east respiratory syndrome (MERS) in 2012, and, in fact, has not been eliminated yet^74^. The ongoing SARS-CoV-2 (a β-coronavirus) led COVID-19 broke out in 2019 and has led to a worldwide catastrophe in the form of a pandemic killing millions of people coupled with causing devastating worst form of health outcomes in millions more globally^75^. The SARS-CoV-2, and its multiple emerging variants, and sub-variants are still a threat to the public health worldwide. We know that SARS-CoV-2 infection depends on ACE2 and TMPRSS2, the host cell’s factors, and hence can be successfully blocked by clinically proven inhibitors; however, our findings might help establish options for the prevention, and treatment, and to lessen the damaging viral effects on human health especially in the vulnerable people since unlike other SARS coronaviruses, the novel SARS-CoV-2 has proven to be highly deleterious to people, especially the ones with underlying co-morbidities/conditions such as diabetes, cardiovascular, renal, and respiratory disease conditions. Understanding that the ACE2 is the coreceptor for the coronavirus has thus prompted new therapeutic approaches to block the ACE2 enzyme or at least reduce its expression to prevent the entry of the SARS-CoV-2 into the host cells.

ACE2 being a key enzymatic component of the renin-angiotensin-aldosterone system (RAAS), it degrades the ANG II, a peptide with multiple actions that is known to promote cardiovascular disease (CVD), and generates the Ang-(1–7), which, in turn, antagonizes the effects of ANG II. The literature suggests that blocking the RAAS by ACE inhibitors; ANG II type 1 receptor antagonists, and mineralocorticoid antagonists, as well as statins, enhance the ACE2 that, in part, contributes to the benefit of these regimens. Since patients with hypertension (HTN) or other CVDs are routinely treated with the RAAS blockers and statins, some concerns have arisen regarding whether these patients are at a greater risk for the SARS-CoV-2 infection, and whether the RAAS and statin therapy should be subjected to re-evaluation, and also the consequences of RAAS blockade to COVID-19-related complications, such as the respiratory diseases^24^.

**Fig. 4:**
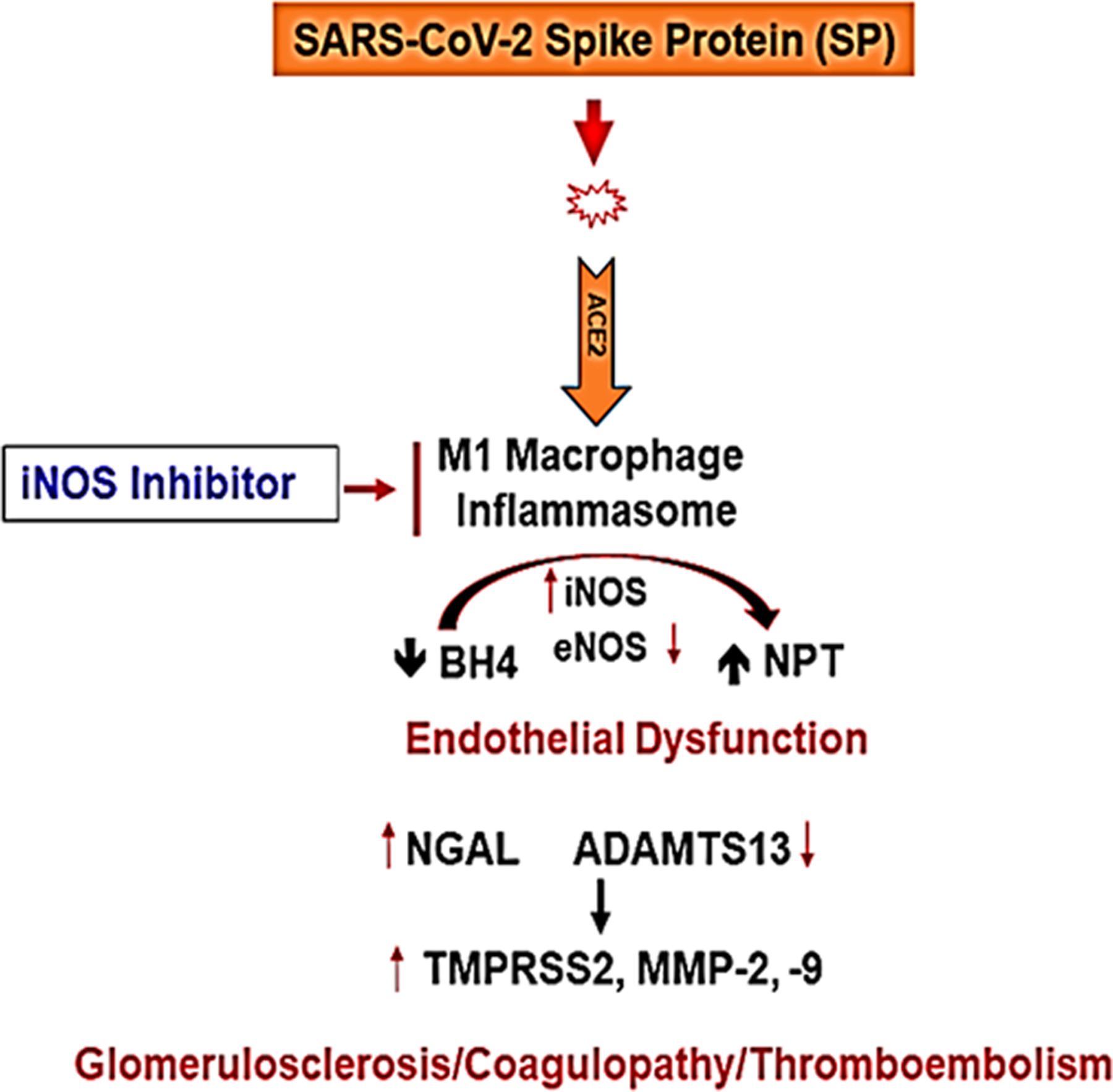
A Schematic representation depicting the SARS-CoV-2 spike protein (SP) induced glomerulosclerosis, coagulopathy, and thromboembolism in the COVID-19 patients. Understanding the pathogenesis of SARS-CoV-2 induced endothelial dysfunction and glomerulosclerosis. The schematic illustrates key molecular players in the pathogenesis of SARS-CoV-2-associated endothelial dysfunction and glomerulosclerosis. The virus enters host cells via the interaction between its spike protein (SP) and the ACE2 receptor, prominently expressed on endothelial cells. This interaction triggers a cascade of events leading to endothelial dysfunction. M1 macrophages are activated and release various factors, including inducible nitric oxide synthase (iNOS), exacerbating endothelial dysfunction. Administration of iNOS inhibitors shows promise in mitigating this effect. In addition, endothelial nitric oxide synthase (eNOS) activity is modulated by factors such as BH4, influencing nitric oxide production. The neutrophil gelatinase-associated lipocalin (NPT) and a disintegrin and metalloproteinase with thrombospondin motifs 13 (ADAMTS13) play crucial roles in the regulation of coagulopathy and thromboembolism. They are key factors in maintaining the delicate balance between pro-coagulant and anti-coagulant states. The transmembrane serine protease 2 (TMPRSS2) facilitates viral entry into host cells, further contributing to endothelial damage. Matrix Metalloproteinases (MMPs) like MMP-2 and MMP-9 are involved in tissue remodeling processes and are implicated in the development of glomerulosclerosis. Together, these molecular interactions culminate in a complex interplay leading to endothelial dysfunction, coagulopathy, and glomerulosclerosis, which are central features of SARS-CoV-2-induced vascular pathology.

Others have shown the role of MMPs, ADAMTS13 in COVID-19^76–78^. We determined that SP causes MMPs activation and in the hACE2 mice the activation was robust than the untreated control mice. The levels of serum MMP-9 were increased by SP but interestingly, the levels of ADAMTS13 (e.g., the decreased level is marker of venous thromboembolism, VTE) was decreased by SP injection in hACE2 mice, when compared with untreated hACE2 mice (Fig. 1). These are novel findings and we have been recognized for development and demonstration of their usefulness in highlighting the tissue remodeling phenomenon^79^. Others have shown the role of growth arrest and DNA damage-inducible 45beta (GADD45beta) in the stress response, cell cycle arrest, in apoptosis, and embryonic growth plate and its significance as an essential mediator of the matrix metalloproteinase-13 (MMP-13) expression during terminal chondrocyte differentiation^80^. We observed robust expression of MMP-13 as shown in the casein gel zymography after the SARS-CoV-2 spike protein administration (Fig. 1). Although primary cause of morbidity and mortality of COVID-19 infection is though the venous thromboembolism (VTE); however, the mechanisms are largely unknown. It is known though that CAC is distinct than the usual coagulopathy and thromboembolism, as discussed elsewhere in this manuscript. The levels of TMPRSS2, NGAL, ADAMTS13, MMP-2, and −9 were successfully measured by zymography and the Western blot analysis. The treatment with AG normalized these levels. Further, the treatment with iNOS blocker mitigated the pro-inflammatory mediated thromboembolism post administration of SP. Our results conclude that the activation of inflammatory M1 macrophage and iNOS increases the NPT thus leading to oxidative/peroxynitrite stress, increased NGAL, MMP-9 and transmembrane serine proteinase S2 (TMPRSS2) and decreases ADAMTS13 which corroborates the findings reported by others recently^42^. This causes vascular injury, coagulopathy, venous thromboembolism (VTE), and vascular leakage that together led to parenchymal injury and CKD during COVID-19 as schematized in Fig. 4.

The spike protein (SP) induces iNOS production by the inflammatory M1 macrophages that sequester all the available tetrahydrobiopterin (BH4) and generate neopterin (NPT) and peroxinitrite leading to the activation of serine, and metalloproteinases, thus instigating the renal glomerulosclerosis, vascular coagulopathy, and VTE. The treatment with iNOS inhibitor mitigates the debilitating effects on SARS-CoV-2 spike protein (SP) in the host. It is noteworthy that biological sex is increasingly recognized as a critical determinant of health and disease, particularly relevant to the COVID-19 pandemic caused by the SARS-CoV-2 coronavirus. Epidemiological data and observational reports from both the original SARS epidemic and the most recent COVID-19 pandemic have a common feature: males are more likely to exhibit enhanced disease severity and mortality than females. Sex differences in cardiovascular disease and COVID-19 share mechanistic foundations, namely, the involvement of both the innate immune system and the canonical renin-angiotensin system (RAS). Immunological differences suggest that females mount a rapid and aggressive innate immune response, and the attenuated antiviral response in males may confer enhanced susceptibility to severe disease. Furthermore, the angiotensin-converting enzyme 2 (ACE2) is involved in disease pathogenesis in cardiovascular disease and COVID-19, either to serve as a protective mechanism by deactivating the RAS or as the receptor for viral entry, respectively. Loss of membrane ACE2 and a corresponding increase in plasma ACE2 are associated with worsened cardiovascular disease outcomes, a mechanism attributed to a disintegrin and metalloproteinase (ADAM17). SARS-CoV-2 infection also leads to ADAM17 activation, a positive feedback cycle that exacerbates ACE2 loss. Therefore, the relationship between cardiovascular disease and COVID-19 is critically dependent on the loss of membrane ACE2 by ADAM17-mediated proteolytic cleavage.

As we slowly emerge from the ongoing crisis, it is anticipated that there should be renewed efforts for true multi-disciplinary research for conducting integrated basic, clinical, and translational studies aimed at increasing the existing knowledge toward understanding how both the current COVID-19 crisis and the future health threats that can potentially affect both the healthy subjects and people with chronic disease conditions, such as diabetes. We remain hopeful that funding this kind of research in the above targeted areas will certainly contribute greatly to solving the paucity of data that currently exists for SARS-CoV-2 led COVID-19. This will also assist in understanding the mechanisms that could potentially modulate the viral pathogenesis. Critical data generated at this time will be extremely useful when informing on future disease preventive, and therapeutic measures.

In summary, although and comprehensive analysis of the pathways influenced by cytokines, orchestrated in response to the virus or its components such the spike protein (SP), could help unravel the COVID-19 disease mechanism(s) underpinning the development of coagulopathy in COVID-19 (CAC). This meticulous dissection not only sheds light on the underpinnings of the condition but also affords us a panoramic perspective of the trajectory that the disease takes as it unfolds within the human body. The role of cytokines in mediating these cascades of events emerges as a crucial focal point, where the immune system and coagulation pathways cross paths in a complex choreography. Again, by meticulously deciphering these pathways, we can assemble a coherent puzzle that captures the holistic essence of disease progression in COVID-19. That kind of comprehension would hold the potential to uncover novel insights, guide clinical interventions, and ultimately, refine our approach to managing the acute phase of the SARS-CoV-2 infection. In essence, delving into the intricate interplay between the inflammation evoked by COVID-19 and the intricate coagulation dynamics exhibited by the patients during the acute stage will allow us to piece together a multidimensional understanding of the disease’s evolution. This understanding, amplified by a comprehensive assessment of the cytokine-driven avenues that contribute to coagulopathy, shall be able to pave the way for a more enlightened approach to managing and mitigating the impact of this complex course of the COVID-19 disease. Through the lens of these insights, we can navigate the complexity driven by the enigma of COVID-19 associated coagulopathy or CAC in the vulnerable patients that remain prone to infection by the emerging SARS-CoV-2 variants, and subvariants that still pause a great danger to aging population, and the immunocompromised individuals.

## METHODS

### Study design and ethics

All reagents were obtained from standard sources. The WT and hACE2 mice were obtained from the Jackson Laboratory. The mice were bred at the University of Louisville School of Medicine transgenic, and knockout rodent core facility. Male and female mice of 10–12 wk were bred and maintained per procedures from the Jackson Laboratory in a 12:12 h light-dark cycle environment. The mice had free access to food and water. In the hACE2 and wild type (WT) mice, SP was administered intra-renally in the cortex, as per the following procedure. Briefly, the abdominal cavity was opened, and kidneys were exteriorized. The control mice were given the sterile buffer. To determine the role of iNOS, mice received iNOS inhibitor (Aminoguanidine: AG 100 mg/kg, intraperitoneally; i.p.) by osmotic mini pump for 4 weeks. At 2-, 4-, and 6-days and for post SP administered at 2, 3 and 4-wks. Samples such as serum and the urine, and feces were collected using the metabolic cages. Renal cortex TMPRSS2, NGAL, and ADAMTS13 were measured by Western blotting and zymography techniques (as described below). The mice were treated with and without iNOS inhibitor AG and its effects were studied. Based on our previous study, and the work done by others, the dose of AG used in our experiments was was100 mg/kg via an’ osmotic minipump delivery (Alzet model 1004) for 28 days^81^. The experiments helped us determine whether the SP administration was able to induce iNOS, and whether SP will be able to instigate the vascular dysfunction that is supposed to be mediated though the interaction of macrophages, neutrophils, and the endothelial cells, thus setting a stage of vascular thromboembolic events in the humanized ACE2 mice (hACE2).

The experimental animal protocol was duly approved on 12/01/2020 by the University of Louisville School of Medicine, Louisville, Kentucky, USA, Institutional Animal Care and Use Committee (IACUC); via the Animal Welfare Assurance Number A3586–01. After completion of the experiments, the animals (mice) were euthanized humanely using an appropriate ‘deep anesthesia’ dosage of tribromoethanol (TBE). The method is consistent with the recommendations of the “Panel on Euthanasia of the American Veterinary Medical Association (AVMA)”.

### Western blotting for protein detection

Antibody and the sterile buffer were obtained from obtained from standard sources, as mentioned above. The tissue protein was isolated using protein extraction buffer (RIPA lysis buffer, protease inhibitor cocktail and PMSF). Lysates were spun in extraction buffer for 12 h and then centrifuged at 12,000 × *g* for 15 min. Supernatant was transferred to new tubes and protein concentrations were analyzed via Bradford protein estimation assay. Samples were run on a 10/12% sodium dodecyl sulfate (SDS)-polyacrylamide gel with Tris–glycine SDS buffer. The gel was transferred electrophoretically overnight onto a PVDF membrane at 4 °C. The membrane was blocked with a 5% milk solution for 1 h. The primary antibody was diluted at a concentration 1:1000 in TBST and incubated on the polyvinylidene (PVDF) membrane overnight. The membrane was washed in TBST solution four times and then incubated with secondary HRP conjugated antibody solution for 1 h at room temperature. Four TBST washing steps followed before membrane was developed using a chemiluminescent substrate in a Bio-Rad Chemidoc (Hercules, Calif.). Band intensity was determined using densitometry analysis. GAPDH was used to normalize protein loading. Equal amounts of total protein (50 μg) were resolved on SDS-PAGE and transferred to PVDF membrane. The membrane was probed overnight at 4 °C with primary antibody followed by 2 h incubation in secondary antibody. The signal capturing was done with the Bio-Rad ChemiDoc XRS+ system and Image Lab software (Bio-Rad, Hercules, CA). The relative optical density of protein band(s) was analyzed using gel software Image Lab 3.0. The membrane was stripped and re-probed with GAPDH as a loading control.

### Zymography for the measurement of the MMPs activities

The zymographic analyses were performed on sera samples and the tissue sample (kidney) homogenates, using 1% gelatin in the gels, as described^82–84^. Samples were prepared and were electrophoretically resolved on 7.5% SDS-PAGE containing 1.5% gelatin as a substrate. In the end, the gels were incubated in renaturation buffer (2.5% Triton X-100) for 30 min to remove SDS, rinsed in distilled water, and then incubated for 24 h at 37 °C in a water bath in an activation buffer (50 mmol/L Tris–HCl, pH 7.4, and 5 mmol/L CaCl_2_). For reverse zymography, 10 μL of active collagenase was added to the active buffer. The gels were stained with the Coomassie R-250 brilliant blue dye for 1 h and then destained them with the destaining buffer (10% acetic acid, 10% methanol, and 80% distilled water). The clear digested regions representing MMPs activities as accessed by running the prestained molecular weight markers were quantified densitometrically using the Un-Scan-It software (Silk Scientific Inc., Orem, Utah, USA).

### Statistical analyses

The data from experimental mice groups were collected, and statistically analyzed using the GraphPad Prism 9.0 (GraphPad Software, United States). Multiple comparisons were performed using one-way ANOVA with Bonferroni, as appropriate to analyze the difference between the groups, including a Tukey’s post hoc analysis for the groups’ comparison. The comparisons between two groups were performed by unpaired Student’s *t* test. The *p < 0.05 was regarded as statistically significant. The data are reported as mean ± SEM, and error bars indicate SEM, *n* = 3–5 animals (mice)/group.

## Data Availability

The datasets generated, and analyzed during this study are available upon request as per the data sharing policies of NIH.
